# Investigating the Consistency of Negative BOLD Responses to Combinations of Visual, Auditory, and Somatosensory Stimuli and Their Modulation by the Level of Task Demand

**DOI:** 10.1002/hbm.70177

**Published:** 2025-03-06

**Authors:** Wilf Nelson, Stephen D. Mayhew

**Affiliations:** ^1^ Centre for Human Brain Health (CHBH), School of Psychology University of Birmingham Birmingham UK; ^2^ Institute of Health and Neurodevelopment (IHN) and School of Psychology Aston University Birmingham UK

**Keywords:** cognitive load, cross modal, deactivation, fMRI, inhibition, NBR, negative BOLD response, sensory

## Abstract

Negative BOLD fMRI responses (NBR) occur commonly in sensory cortex and default mode network regions but remain poorly utilized as a marker of brain function due to an incomplete understanding. To better understand how NBR manifest across the brain, compare between different sensory stimuli and how they are modulated by changes in task demand, we recorded fMRI during trials of visual, auditory, or somatosensory stimulation, delivered either alone or in concurrent pairs. Twenty young‐adult participants were cued to attend to a single modality and detect targets in each trial. We found that NBR were consistently induced in all non‐task‐relevant primary sensory cortices and default mode regions during all stimuli. NBR were observed within the stimulated modality, in the cortex ipsilateral to the stimulus; as well as cross‐modal responses bilaterally within the cortex of an unstimulated sensory modality. The NBR regions showed high spatial overlap with the primary sensory positive BOLD response (PBR) of the stimulated modality. The NBR occurred in spatially comparable regions across different modality stimuli such that the peak voxel location and spatial extent were comparable between within and cross‐modal NBRs. Some specific differences were seen, such as stronger magnitude sensorimotor NBR to somatosensory stimuli than to visual or auditory. No significant relationships were found between subjects' PBR and NBR magnitude, but significant linear correlations were observed between NBRs indicating that subjects with high magnitude NBR within one sensory modality also displayed high magnitude cross‐modal NBR in a different modality. These findings suggest that cortical NBR are largely consistent between different sensory stimuli but also contain stimulus‐specific variability in magnitude and spatial extent. Finally, positive BOLD responses were stronger to dual stimuli in all contralateral primary sensory regions, whilst NBR were slightly increased in specific regions of ipsilateral visual and sensorimotor cortex. This finding suggests a strong contribution to NBR from bottom‐up stimulus input that was further modulated by attention during dual conditions and that NBR is driven by a combination of bottom‐up and top‐down influences whereby contributions to its generation arise from both feed‐forward signals from subcortical or activated sensory regions and feedback mechanisms such as higher‐level attentional control.

## Graphical abstract

An fMRI study investigating deactivations both within and between sensory cortices during combinations of visual, auditory, and somatosensory stimuli. We study the similarities and differences in the spatial extent of deactivations and how they are modulated by increased attentional demand when processing multiple stimuli.

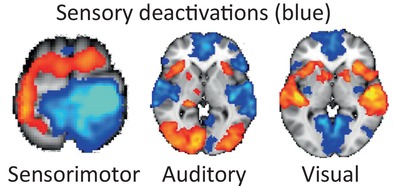

**Summary**

- Negative BOLD responses (NBRs) were studied during visual, auditory, and somatosensory stimuli delivered alone or in pairs.
- NBRs were observed in all unstimulated sensory cortical areas and the default mode network and occurred in a broadly consistent manner across conditions.
- Some areas of significantly increased NBR magnitude to paired stimuli were observed in the visual and sensorimotor cortex, suggesting an additional top‐down attentional modulation.
- No significant relationships were found between the subject's PBR and NBR magnitudes, but significant linear correlations were observed between NBRs, indicating that subjects with high magnitude intra‐modal NBR also displayed high magnitude cross‐modal NBR.

## 1 Introduction

Functional magnetic resonance imaging (fMRI) is a widely used non‐invasive neuroimaging technique for investigating human brain function. Increases in neuronal activity are commonly inferred from increases in the blood oxygenation‐level dependent (BOLD) signal and used to investigate the brain regions recruited by task processing (Ekstrom 2010; Logothetis 2002; Viswanathan and Freeman 2007; Zhang et al. 2020). Such positive BOLD responses (PBRs) represent only an indirect, haemodynamic measure of brain function, comprising an increase in the local oxyhaemoglobin content of the blood, which occurs from an increase in cerebral blood flow (CBF) and volume (CBV) to compensate for increased metabolism of oxygen due to increased peri‐synaptic neural activity (Buxton and Frank 1997; Hoge et al. 1999; Logothetis 2008).

Task‐induced decreases in BOLD signal relative to pre‐stimulus baseline, known as negative BOLD Responses (NBR), are also commonly observed during most tasks but are not fully exploited for functional imaging. Cortical NBRs arise primarily in two forms: in the sensory cortex representation of unstimulated areas of the sensory field (Allison et al. 2000; Shmuel et al. 2002); and in the default mode network (DMN) which is typically comprised of the posterior cingulate cortex (PCC), bilateral intra‐parietal lobe (IPL), and medial prefrontal cortex (mPFC) (Buckner et al. 2008; Raichle et al. 2001). Sensory NBRs are the main topic of the present study and can be separated into two distinct types: (1) intra‐modal (IM) NBRs such as in visual cortex areas unstimulated by partial visual field stimuli (Bressler et al. 2007; Fracasso et al. 2018; Gouws et al. 2014; He et al. 2022; Mayhew et al. 2013; Wade and Rowland 2010; Wilson et al. 2020) or the sensorimotor cortex ipsilateral to unilateral limb movements and somatosensory stimulation (Hlushchuk and Hari 2006; Kastrup et al. 2008; Klingner et al. 2010; Klingner et al. 2010; Klingner, Ebenau, et al. 2011; Mullinger et al. 2014; Newton et al. 2005); or (2) cross‐modal (CM) NBRs such as in auditory cortex during visual stimulation and vice versa (Laurienti et al. 2002; Mayhew et al. 2013; Mozolic et al. 2008; Wilson et al. 2019). NBRs can arise in other specific circumstances such as due to blood volume effects around large cerebral veins and ventricles (Bianciardi et al. 2011; Bright et al. 2014; Puckett et al. 2014) and have been reported in rat hippocampus arising from increased neural activity and unusual neurovascular coupling (Schridde et al. 2008). Such studies evidence that NBRs can arise from various combinations of changes in the many physiological components of the BOLD signal, which contributes to uncertainty in the understanding of NBRs and limits their functional application.

The generating mechanisms of cortical NBR have been widely debated and can be broadly divided into: vascular mechanisms, such as reductions in CBF due to blood steal or blood sharing (Harel et al. 2002; Hu and Huang 2015); or neuro‐metabolic mechanisms whereby local neural activity and metabolism are decreased due to increased local inhibition or reduced afferent excitation (Devor et al. 2007; Shmuel et al. 2002; Smith et al. 2004). Evidence for a neural contribution has come from observations of decreases in local field potential activity in visual NBR regions in primates (Shmuel et al. 2006) and sensorimotor NBR regions in rats (Boorman et al. 2010; Boorman et al. 2015; Lee et al. 2019). In humans, correlations between NBR and 8–13 Hz alpha‐frequency EEG power have been observed (Mullinger et al. 2014; Wilson et al. 2019) as well as decreases in oxygen metabolism in human motor and visual cortex NBR regions (Mullinger et al. 2014; Pasley et al. 2007; Shmuel et al. 2002; Stefanovic et al. 2004). A consensus is emerging that sensory IM NBR reflects, at least in part, a measure of cortical inhibition (Sten et al. 2017), although not simply reflecting the inverse of the PBR but involving different neurovascular coupling mechanisms (Devi et al. 2022; Huber et al. 2014; Mayhew et al. 2014; Mullinger et al. 2014; Rosa et al. 2021).

Behavioural evidence is sparse, but ipsilateral NBR induced by electrical median nerve stimulation has been shown to correlate with participants' perception threshold for currents delivered to their unstimulated hand (Kastrup et al. 2008; Schafer et al. 2012). NBR has been shown to be retinotopically organised (Bressler et al. 2007) and to differentiate between conditions of visual imagery and perception (Amedi et al. 2005). In addition, evidence has suggested that visual perception can be suppressed by interfering stimuli from both auditory and somatosensory domains (Hidaka and Ide 2015; Ide and Hidaka 2013) and linked to visual cortex NBR (Ide et al. 2016). These studies suggest that the NBR possesses some functional relevance, but exactly what purpose NBR serves in brain processing remains unknown.

A key approach to investigating the origins and functional relevance of NBR is to study experimental factors that modulate the response, such as changes in sensory input, task demands, and attention. Sensory NBR has been shown to increase in magnitude with stimulus intensity (Klingner et al. 2010; Shmuel et al. 2002) and task difficulty (Hairston et al. 2008). In addition, the allocation of spatial attention has been shown to modulate both PBR and NBR magnitude (Gouws et al. 2014; Hairston et al. 2008; He et al. 2022; Mozolic et al. 2008; Tootell et al. 1998). Attentional mechanisms have been shown by both fMRI and electrophysiological studies to prioritise processing of one spatial location or sensory modality to improve task performance, whilst non‐task‐relevant regions are suppressed to minimise potential distraction and interference (Corbetta and Shulman 2002; Mazaheri et al. 2014; Mazaheri and Jensen 2010). Consequently, NBR may arise due to the withdrawal of attention away from the sensory regions that are not receiving stimulus input in order to focus resources and prioritise cortical processing of the task. The current study aims to investigate this question by studying how the magnitudes of PBR and NBR are modulated by changes in task attentional demand between uni‐sensory and dual‐sensory modality stimulus conditions. Participants performed detection of infrequent targets within a train of stimuli, attending only to the target stimulus modality and ignoring other modalities in order to perform the task.

We recorded BOLD responses during unilateral visual, auditory, and somatosensory stimulation, with stimulus modalities delivered either alone or concurrently when one was paired with another. These stimuli allowed a thorough investigation of different NBRs as they induced IM and CM NBRs in visual, auditory, and somatosensory cortices as well as in the DMN. We studied multiple instances of NBR, as the majority of previous work pertains to IM NBR, and therefore it was important to investigate whether IM and CM NBRs exhibited similar response properties or shared similar mechanisms, and also whether they are distinct from those underlying DMN NBR.

The study has two primary aims: first, to investigate the consistency of NBR between different sensory modality stimulus conditions; and second, to investigate to what extent NBR is modulated by the increase in attentional demand associated with increasing from single‐ to dual‐modality stimuli. In addressing these aims, we will investigate the following five questions: (1) Do NBR occur similarly in unstimulated sensory cortex during different sensory stimuli? (2) How do the spatial locations of IM and CM NBR compare to those of the PBR and to each other? (3) How do the magnitudes of the IM and CM NBR compare across conditions? (4) What is the relationship between the responses; do subjects that show strong IM NBR also show strong PBR or CM NBR? (5) Do dual modality stimuli cause greater magnitude NBR than single modality stimuli? Our task modulation involves increasing the number of stimulated sensory modalities and therefore the cognitive load and attentional allocation required to perform the task. We compared how the magnitude and spatial extent of IM and CM NBR differed between these conditions. Under the hypothesis that the addition of a second stimulus modality both withdraws attention even further from the unstimulated sensory cortex and provides an additional NBR generating sensory input, we expect to find that both intra‐modal and cross‐modal NBR magnitude is increased in dual stimulation conditions compared to unimodal.

## 2 Methods

Twenty healthy, adult participants (9 males, aged 20–29 years, mean 24 ± 2.3 years) were recruited from the University of Birmingham student population. All participants were dominantly right‐handed. The study was conducted with the approval of the University of Birmingham Ethics Board, and informed consent was obtained from all subjects before their participation.

fMRI was used to record BOLD responses to 6 s periods of visual, auditory, and somatosensory stimulation. Sensory stimuli were either single‐modality (e.g., visual) or dual‐modality, where two were delivered concurrently (e.g., visual and auditory). The experiment featured six conditions in total. Three conditions had single‐modality stimulation: visual (V), auditory (A) and somatosensory (S); and three conditions had dual‐modality stimulation: visual and auditory (VA), auditory and somatosensory (AS), and visual and somatosensory (VS). The paradigm ensured there was always at least one non‐task‐relevant sensory region, and most often two regions, within which we could investigate the NBR, see Table 1. In each trial, participants were cued to attend and detect the number of targets (0, 1, or 2) presented in one sensory modality. The target modality could be presented alone (V, A, or S conditions) or concurrent with another modality in the dual conditions (VA, AS, and VS). Targets were only presented in one modality per trial. The intention of the dual‐modality trials was not to act as a distractor to target detection, but to further increase the need to divert attention away from the unstimulated modality and modulate the NBR.

**TABLE 1:** The six stimulus conditions used in the experiment and the cortical areas that were expected to show PBR and NBR, respectively.

Condition	Stimulated sensory modality	Expected PBR regions	Expected intra modal (IM) NBR regions	Expected cross modal (CM) NBR regions
A	Auditory	Bilateral auditory cortex	None	Bilateral visual and somatosensory cortices
S	Somatosensory	Contralateral somatosensory cortex	Ipsilateral somatosensory cortex	Bilateral visual and auditory cortices
V	Visual	Contralateral visual cortex	Ipsilateral visual cortex	Bilateral auditory and somatosensory cortices
AS	Auditory and somatosensory	Bilateral auditory and contralateral somatosensory cortex	Ipsilateral somatosensory cortex	Bilateral visual
VA	Visual and auditory	Bilateral auditory and contralateral visual cortex	Ipsilateral visual cortex	Bilateral somatosensory
VS	Visual and somatosensory	Contralateral visual and somatosensory cortex	Ipsilateral visual and somatosensory cortices	Bilateral auditory cortex

The visual stimulus was a black‐ and‐white radial checkerboard of 100% contrast, with pattern reversal at 7 Hz. The checkerboard was wedge‐shaped, subtending 45° of visual angle and displayed in the lower quadrant of the right hemifield. The auditory stimulus was a 7 Hz train of pure tone (1 kHz) beeps delivered to the right ear. The somatosensory stimulus was electrical median nerve stimulation (MNS) delivered at 7 Hz via two electrodes placed on the inside of the right wrist. The MNS was delivered using a Digitimer DS7A stimulator (0.5 ms square wave pulses) with the current amplitude set just above the individual's motor threshold so as to cause a small, involuntary thumb distension. In every trial, participants detected targets presented in a single, attended sensory modality. Targets were never delivered in more than one modality. The targets were brief, deviant stimuli presented amongst the train of standard 7 Hz stimuli, in the form of one of the following in attended auditory trials, a higher pitch (+50 Hz) of an auditory tone; in attended somatosensory trials, a larger temporal interval (+30 ms) between MNS pulses; in attended visual trials, a deviant contrast in a single checkerboard reversal (80% instead of 100% contrast).

**FIGURE 1:**
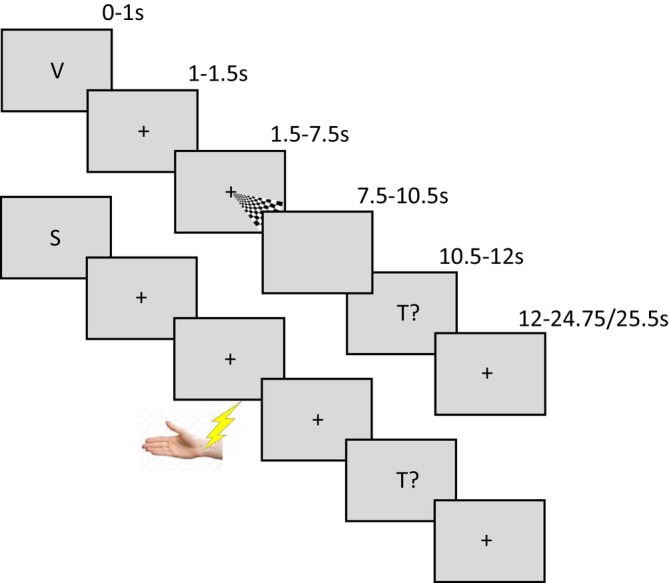
Timeline of an example trial for V (upper) and AS (lower) conditions. All trials began with a 1 s display of a letter (either V, A or S) cueing to the one sensory modality that was to be attended for target detection during the upcoming stimulation. Cues were followed by a 500 ms display of a central fixation cross before a 6 s period of stimulation. Stimulation was at 7 Hz and could include either 0, 1, or 2 deviant targets. A 3 s period of resting fixation separated the end of stimulation from the 1.5 s presentation of T? which cued the subjects to report how many targets they detected by pressing one of three buttons. Finally, an inter‐stimulus interval of 12.75 or 13.5 s occurred before the start of the next trial.

Throughout the experiment, a grey screen was presented with a central black fixation cross. An experimental trial consisted of the following structure and is also illustrated in Figure 1:

- A 1 s cue period, during which a centrally displayed capital letter (V, S or A. 2° of visual angle, 50% contrast) indicated the modality of the subsequent stimulus to which the subject should attend and detect targets.
- A 1 s period of resting fixation.
- A 6 s period of sensory stimulation. Stimuli were presented throughout the whole period and contained either 0, 1, or 2 target stimuli in the cued modality only.
- A 3 s period of resting fixation.
- A 1.5 s response period where ‘T?’ was displayed in the centre of the screen. Participants' responses were given by pressing one of three keys on an MRI compatible button box corresponding to the detection of either 0, 1, or 2 targets in that trial.
- Finally, a 12.5 or 13.5 s period of resting fixation acted as a baseline period. A one‐second jitter was employed equally over conditions to provide better sampling of the BOLD haemodynamic response.

During each trial, the subject's task was to attend to the cued modality and detect any targets presented in it, whilst ignoring any other stimuli. The subject only reported the number of detected targets during the T? response period after the stimulation had ceased. This temporally separated the response from the stimulation in an effort to minimise the contamination of somatosensory BOLD responses by motor responses to button pressing. Data collection was organised into four runs, each lasting 13 min. Five trials of each condition were delivered in each run, presented in a pseudo‐randomised order, totalling 20 trials per condition, per subject. In each condition there were four trials with 0 targets, eight trials with 1 target, and eight trials with 2 targets. The attended modality was counterbalanced amongst dual trials, that is, for the VS condition there were an equal number of attend‐to‐V (10) and attend‐to‐S (10) trials per subject. Immediately prior to scanning subjects were given a practise run of the task to ensure they understood the paradigm and could perform the target detection reliably enough to score above 66% accuracy in all conditions.

A 3 T Philips scanner (Philips Medical Systems, Best, Netherlands) was used to acquire MRI data with a transmit body coil and 32‐channel head coil. We used gradient‐echo EPI to acquire T2*‐weighted BOLD fMRI data with multiband factor = 2 and SENSE = 2.3, TR = 1.5, TE = 38 ms, flip angle = 70°, FOV = 96 × 96, and 40 slices with 0.2 mm slice gap and a voxel size of 2.5 mm isotropic providing whole head coverage. Cardiac and respiratory signals were recorded using the scanner's inbuilt pulse oximeter (PPU) and bellows. A whole‐head T_1_‐weighted anatomical image (TR = 2000 ms, TE = 2 ms, TI = 880 ms, flip angle = 8°, FOV = 256 × 256, 1 mm isotropic resolution) was acquired between the second and third task runs to facilitate image co‐registration.

### 2.1 Behavioural Data Analysis

Behavioural data were analysed to calculate the accuracy of target detection (% correct trials) for each condition. Due to the paradigm's delay period, we did not compute reaction times. Trials were only considered correct if all targets were correctly responded to; therefore, trials with two targets but only one detection were classed as incorrect. Additionally, one‐way ANOVAs were conducted in SPSS (ww.IBM.com) to compare performance during single modality conditions against dual conditions that shared that modality. Specifically, three ANOVAs were used to compare accuracy during the following: V, VS, and VA; A, VA, and AS; S, AS, and VS.

### 2.2 fMRI Data Analysis

fMRI data were preprocessed and analysed in FSL v6.01 (http://www.fmrib.ox.ac.uk/fsl). The BOLD data were motion‐corrected using FLIRT (Jenkinson et al. 2002; Jenkinson and Smith 2001), high‐pass temporally filtered (> 0.01 Hz), spatially smoothed using a 5 mm FWHM Gaussian kernel, and spatially normalised by coregistration to the subject's T1 anatomical (7 DOF) which was itself coregistered to the 2 mm MNI template (12 DOF) using FLIRT.

The PPU and respiratory data of each subject were input to the PhysIO toolbox (Kasper et al. 2017) which was used to calculate time course regressors that modeled variability in physiological noise. A total of three cardiac and four respiratory terms were used, along with 1 interaction term, to create RETROICOR style regressors (Glover et al. 2000). Also, the respiration per volume time (RVT) (Birn et al. 2008) and heart‐rate variability (Chang et al. 2009) regressors were modeled. These regressors were included, as covariates of no interest, in the first‐level general linear model (GLM) design matrix, along with the six main parameters of head motion output by MCFLIRT.

First‐level GLM analyses were performed using FSL FEAT v6.01 for each run from each participant. Each of the six conditions was modelled as a separate regressor using the respective stimulus timings. In addition, two separate regressors were used to model the presentation of the pre‐stimulus cue and the subject's button press during the response period. All trials were modelled. We did not discard incorrect trials as the study's primary motivation was to investigate BOLD response differences between single and dual conditions, and we prioritised analysing all available data to maximise statistical power. As all participants subjectively informed us, when asked after the session, that they had been able to attend as instructed, and given that performance was consistently high (> 75%, see Table 2) we feel that attention was appropriately allocated to the task.

Each regressor was convolved with a double‐gamma haemodynamic response function, and their temporal derivatives were also included in the design matrix. Both positive and negative contrasts were used to identify regions of PBR and NBR, respectively. Contrasts were compiled across all four runs using fixed effects to give the mean response per condition and per participant at the second level. The third‐level analysis was then used to calculate group‐level results, using mixed‐effects FLAME 1 for PBR and fixed effects for NBR due to the lower signal‐to‐noise ratio of that response. All group‐level maps were cluster corrected at *p* < 0.05, Z > 3.1. NBR response amplitude is substantially lower than that of the PBR and can be as little as 25%–50% of the PBR percentage signal change from baseline (Kastrup et al. 2008; Klingner et al. 2010; Klingner, Huonker, et al. 2011; Shmuel et al. 2002; Wilson et al. 2020). Therefore, a more liberal statistical approach for NBR was used in this work, as has been implemented before (Klingner, Huonker, et al. 2011; Mullinger et al. 2014; Mullinger et al. 2017; Wilson et al. 2019; Wilson et al. 2020).

These third‐level contrasts provided the group main effect of each condition for both PBR and NBR. Further analyses were then conducted to test for differences in response between conditions. We first tested for a general difference between dual and single condition responses, independent of modality, by contrasting the sum of all single conditions against the sum of all dual conditions ([V + A + S] vs. [VA + AS+VS]). Six further 3rd level analyses were then conducted by contrasting each dual condition against each single condition that was part of it (e.g., VS vs. V, VS vs. S, AV vs. A, AV vs. V, AS vs. A, AS vs. S).

### 2.3 ROI Analysis

We performed further analysis to provide a more detailed comparison of response magnitude, extent, and location between IM and CM NBRs. Group‐level masks of bilateral visual, auditory, and sensorimotor cortex obtained from the FSL atlas were used to separately mask group‐level PBR and NBR Z statistics for each of the six conditions. For the DMN, masks of the PCC and mPFC were obtained from the FINDlab atlas (Shirer et al. 2012). These PBR and NBR masks were then applied to the second‐level Z‐statistic maps of each subject to calculate the peak voxel location, the peak Z‐statistic, and the centre of gravity (COG) of the responses to each condition. Also, the mean Z‐statistic of all non‐zero voxels in the mask and the number of non‐zero voxels were calculated.

We then studied the spatial overlap between PBR and NBR and also between IM and CM NBRs in order to evaluate the consistency of NBR regions across stimulus conditions, and also whether NBR occurred in similar regions to those activated (i.e., showed PBR). To allow the most detailed comparison of the NBRs with visual and sensorimotor PBR, we reflected the lateralised PBRs around the *x* = 0 axis to be able to represent them in the ipsilateral cortex as well. Separately for each condition, subjects' second‐level NBR and PBR Z‐statistic maps were binarised and then used to calculate the overlap between responses. Specifically, separately for visual, auditory, and sensorimotor cortices, the conjunction of the respective PBR with the NBR to each condition was computed. The conjunction of IM and CM NBRs was also calculated. As an overall summary measure, the conjunction of all IM NBRs and the conjunction of all CM NBRs were also calculated. Dice coefficients and % overlap (e.g., % of NBR overlapping with PBR) were also computed as summary metrics. These analyses investigated whether spatially similar areas showed NBR in different stimulus contexts because such comparisons will help inform the physiological similarity between the responses.

### 2.4 Studying Relationships Between PBR and NBR

The sensory cortex ROIs were used to extract the beta weight (COPE) from the peak voxel location for PBR, IM NBR, and CM NBR in each condition. COPE stands for contrast of parameter estimate in the FSL software and is the result of multiplying the beta weight by the contrast. We used the COPE, as both PBR and NBR large positive COPE values are associated with a strong model fit (and vice versa) and so when plotting correlations, both can be similarly interpreted. Linear correlations were then computed between the peak beta weight of PBR and each NBR, and between the peak beta weight of IM– NBR and CM –R. We investigated this when responses were induced in different sensory cortices by the same stimulus (e.g., NBR in iV1 vs. NBR S1 during V condition) or when induced in one sensory cortex by different stimuli (e.g., NBR in iV1 during V compared to the S condition). We computed all pairs of correlations, but upon noticing a clear pattern for correlations between IM and CM NBR across different conditions, we summarised the results by separately averaging all IM NBR betas and all CM NBR betas together. For example, the IM NBR in V1 was calculated from the mean across V, VA, and VS conditions; whereas the CM –NBR in V1 was calculated from the mean of across A, S, and AS conditions.

## 3 Results

### 3.1 Behavioural Results

The group mean behavioural data for each condition is shown in Table 2 below. Target detection ranged from a minimum of 73.7% in the S condition to a maximum of 86.5% in the V condition. Performance was highest in the V, A, and VA conditions (82.6%–85.5%). The conditions featuring somatosensory stimulation showed the lowest performance (74.7%–76.3%). All ANOVAs comparing single vs. dual condition accuracy were insignificant, indicating that participants performed equally as well during the dual stimulation conditions as during the single modality conditions; for example, VA performance was highly comparable to V, and VS performance was comparable to S.

**TABLE 2:** Group mean percentage accuracy for each condition with standard error in the mean.

Condition	Group mean accuracy (% trials correct) ± SEM
V	85.5% ± 5.8%
A	82.6% ± 5.5%
S	74.7% ± 5.0%
VA	83.6% ± 5.7%
AS	76.3% ± 4.7%
VS	75.4% ± 4.9%

### 3.2 fMRI Results

**FIGURE 2:**
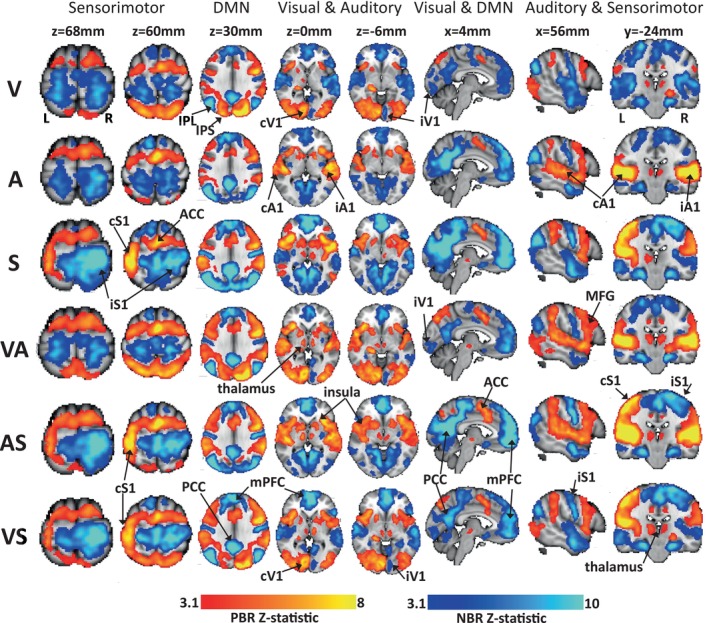
Group main effects of stimulation in each condition. Third‐level PBR (red/yellow) and NBR (blue) are displayed on brain slices chosen to allow comparison of the NBR between conditions in the primary visual (V1), auditory (A1) and somotosensory (S1) cortex as well as the DMN. The left hemisphere is shown on the left of the image. All maps are cluster corrected *p* < 0.05. Rows 1–6 show responses to visual (V), auditory (A), somotosensory (S), visual and auditory (VA), auditory and somatosensory (AS) and visual and somatosensory (VS) conditions respectively. Column heading labels indicate the key regions displayed. Columns 1, 2, 7, and 8 show slices through sensorimotor cortex; columns 3 and 6 show midline slices displaying the DMN; visual cortex is shown in columns 4, 5 and 6; and auditory cortex is shown in columns 4,5,7 and 8. PBR maps are mixed effects, NBR maps are fixed effects, all cluster corrected at *p* < 0.05, *Z* > 3.1. Labels and arrows denote the key regions: A1 = primary auditory cortex; ACC = anterior cingulate cortex; c = contralateral; i = ipsilateral; IPL = intra parietal lobe; IPS = intraparietal sulcus; MFG = medial frontal gyrus; mPFC = medial prefrontal cortex; PCC = posterior cingulate cortex; S1 = primary sensorimotor cortex; V1 = primary visual cortex.

Z‐statistic maps of the group mean PBR (red‐yellow) and NBR (blue) to each condition are displayed in Figure 2, which allows for a detailed comparison of the spatial location and magnitude of responses between conditions across the whole brain. GLM analysis that modeled incorrect trials as confounds returned qualitatively nearly identical statistical maps (not shown), we do not consider the effect of incorrect trials any further.

We now describe the PBR, IM NBR, CM NBR, and DMN NBR results in detail, first starting with a general description of their locations, then describing and comparing them in four key metrics: magnitude, peak voxel location, spatial extent, and degree of spatial overlap.

#### 3.2.1 PBR Locations

PBRs were observed in the primary sensory cortex of the stimulated modalities in each condition. During all conditions, including V or S stimuli, PBR was observed in the contralateral visual (cV1) and sensorimotor (cS1) cortex, respectively. Bilateral PBR was observed in the primary auditory cortex (A1) during A, AS, and AV conditions. Additionally, PBR was observed in the anterior insula cortex, the anterior cingulate cortex (ACC), the medial frontal gyrus (MFG) and the thalamus in all conditions. PBRs were observed in the dorsal parietal cortex, around the intraparietal sulcus (IPS) in all conditions that involved a visual stimulus.

Table 3 contains further ROI metrics of the PBR for each condition to enable comparison between conditions and also with subsequent NBR locations. PBR peak locations and response magnitudes were largely consistent between single and dual modality conditions. Table 3 indicates that the peak magnitude and spatial extent of sensory PBR were similar across all conditions.

**TABLE 3:** PBR peak voxel location (in mm), magnitude (peak and mean Z‐statistic), extent (number of voxels), and centre of gravity (COG, mm) for each condition (V, A, S, VA, AS, and VA) in the contralateral (c, left) hemisphere of the primary visual (V1), auditory (A1) and sensorimotor (S1) cortices.

PBR metric	V	A	S	VA	AS	VS
cV1 peak Z	6.5	—	—	6.7	—	6.8
cV1 peak coordinate	−8, −84, −6	—	—	−8, −88, −6	—	−8, −86, −6
cV1 COG	−28, −82, 2	—	—	−28, −82, 2	—	−28, −82, 4
cV1 mean Z	4.5	—	—	4.7	—	4.6
cV1 extent	4240	—	—	4548	—	4445
cA1 peak Z	—	6.4	—	6.3	6.8	—
cA1 peak coordinate	—	−60, −40, 14	—	−58, −40, 14	−40, −24, 0	—
cA1 COG	—	−51, −21, 8	—	−53, −21, 8	−51, −18, 2	—
cA1 mean Z	—	4.2	—	4.3	4.4	—
cA1 extent	—	3387	—	3264	3737	—
cS1 peak Z	—	—	5.9	—	5.5	5.7
cS1 peak coordinate	—	—	−58, −22, 46	—	−56, −22, 48	−54, −22, 50
cS1 COG	—	—	−46, −26, 50	—	−45, −25, 51	−46, −26, 50
cS1 mean Z	—	—	4.4	—	4.2	4.2
cS1 extent	—	—	3847	—	3566	3381

*Note:* (—) Indicates there was no relevant data for that condition, as no PBR was observed.

#### 3.2.2 NBR Locations

As shown by Figure 2, IM NBR was observed widely in the ipsilateral sensorimotor cortex (iS1) during each of the somatosensory stimulation conditions (S, AS and VS). This IM NBR also included midline sensorimotor regions. During each of the visual stimulation conditions (V, AV, and VS) IM NBR was observed in two clusters in the anterior and posterior ipsilateral visual cortex (iV1). Due to the bilateral auditory PBR during the A, AS, and VA conditions, no IM NBR was observed in the auditory cortex.

For clarity, we consider IM NBR to be those induced in the sensory cortex representing the stimulus modality, for example, in visual cortex IM NBR occurred during V, VA, and VS conditions, and CM NBR occurred during A, S, and AS; and in sensorimotor cortex IM NBR occurred during S, AS, and VS conditions, and CM NBR occurred during V, A, and VA conditions. Whilst the responses induced by dual modality stimuli could be considered a combination of both IM and CM NBR, IM appears to be the dominant factor, as described below.

As shown by Figure 2, CM NBRs were consistently observed bilaterally in all non‐task relevant sensory cortices. CM NBRs were induced bilaterally in primary auditory cortex during V, S, and VS conditions. CM NBRs were also induced bilaterally in sensorimotor cortex during V, A, and VA conditions. CM NBRs were induced bilaterally in primary visual cortex during A, S, and AS conditions. The spatial location of the CM NBRs showed a high degree of overlap with both the IM NBRs and the PBRs induced by other conditions, as discussed further below.

During all conditions, NBR was observed in the PCC, IPL, and mPFC core regions of the DMN, as well as the rostral medial temporal lobe and hippocampus.

Table 4 contains specific ROI metrics that allow further comparison of IM, CM, and DMN NBRs between conditions.

**TABLE 4:** Comparison of IM, CM, and DMN NBR peak voxel location (in mm), magnitude (peak and mean *Z*‐statistic), extent (number of voxels) and centre of gravity (COG, mm) for each condition (V, A, S, VA, AS, and VA). For each of the five ROIs encompassing the key NBR regions, the table displays four parameters of the NBR for each of the six stimulus conditions.

NBR	V	A	S	VA	AS	VS
iV1 peak *Z*	7.1	9.9	15.4	6.7	12.6	5.5
iV1 peak coordinate	6, −86, −8	4, −84, 14	2, −82, 16	6, −86, −8	2, −80, 16	6, −82, −6
iV1 COG	8, −85, −2	4, −78, 3	3, −78, 2	8, −82, −2	7, −80, 2	4, −76, 0
iV1 mean *Z*	3.9	4.9	6.5	3.8	5.9	3.8
iV1 extent	912	2968	5908	993	4763	1133
iA1 peak *Z*	10.9	—	7.6	—	—	8.3
iA1 peak coordinate	42, −14, 10	—	34, −24, 14	—	—	36, −22, 14
iA1 COG	46, −20, 8	—	38, −20, 8	—	—	44, −20, 10
iA1 mean *Z*	6.6	—	4.7	—	—	4.4
iA1 extent	2268	—	1282	—	—	1401
iS1 peak *Z*	11.8	10.2	16.7	10.6	16.3	17.4
iS1 peak coordinate	24, −32, 64	26, −34, 68	30, −32, 66	20, −32, 62	24, −32, 64	24, −32, 62
iS1 COG	2, −28, 60	3, −32, 62	26, −30, 63	2, −29, 60	22, −30, 63	20, −30, 61
iS1 mean *Z*	5.5	4.9	8.2	5.1	8.1	7.8
iS1 extent	6334	5524	8216	5900	8064	7915
PCC peak *Z*	10.9	13.5	17.1	8.8	16.7	9.9
PCC peak coordinate	−6, −52, 34	−2, −58, 28	0, −58, 28	−4, −58, 18	−4, −58, 24	−4, −56, 18
PCC COG	−2, −54, 30	0, −54.27	0, −56, 28	−1, −54, 22	0, −54, 23	−1, −53, 22
PCC mean *Z*	7.7	8.3	12.2	4.9	8.5	5.9
PCC extent	1215	1459	1518	1172	1384	1390
mPFC peak *Z*	11.4	11.9	18.5	10.3	17.9	10.76
mPFC peak coordinate	0, 46, −12	−6, 58, 6	−2, 52, −12	0, 50, −12	0, 52, −14	0, 50, −14
mPFC COG	0, 50, 0	−2, 54, 2	0, 54, −2	1, 54, 0	0, 54, −4	1, 54, −4
mPFC mean *Z*	6.8	6.6	10.1	5.6	8.7	6.3
mPFC extent	4078	4205	4390	4182	4345	4313

*Note:* (—) Indicates there was no relevant data for that condition, as only PBR was observed. Responses were measured from the ipsilateral (I, right) hemisphere of the primary visual (V1), auditory (A1) and somatosensory (S1) cortices as well as the posterior cingulate cortex (PCC) and medial prefrontal cortex (mPFC) of the DMN.

#### 3.2.3 Comparing IM and CM NBRs to Each Other and to the PBR

We now compare the magnitude and spatial extent of responses between conditions. In sensorimotor cortex, the IM NBRs (during S, AS, and VS) displayed the greatest spatial extent (7915–8216 voxels) and magnitude, with all showing peak voxel *Z* > 16. In comparison, the CM‐NBRs (during V, A, and VA) showed peak voxel *Z* = 10.2–11.8 and spatial extent 5524–6334 voxels (Table 4). Both IM NBRs and CM NBRs showed highly consistent spatial extent across conditions. The IM NBR was more strongly lateralised than the bilateral CM NBR, and the IM NBR also showed greater spatial extent in iS1 than the CM NBR. The iS1 NBR peak voxel locations lay in similar areas of the ipsilateral (right) somatosensory gyrus across all six conditions. The NBR COG was also lateralised to the right iS1 for S, AS, and VS conditions but was close to the midline for V, A, and VA, which reflected a more bilateral NBR in those conditions. Comparing Tables 3 and 4 it is evident that the NBR peak voxel and COG in S1 were more dorsal than those of the PBR, with NBR peaks located at 60–68 mm compared to 42‐50 mm for peak PBR voxels. This can also be seen in Figure 2.

In the auditory cortex, only CM NBRs were observed and were very similar in extent (1282–1401 voxels) and magnitude (*Z* = 7.6–8.3) between S and VS conditions. The greatest magnitude (*Z* = 10.9) and most spatially extensive (2268 voxels) CM NBR was induced by the V condition (Table 4). The CM NBR during V was the most widespread bilaterally, encompassing more dorsal areas of the auditory cortex than were observed during S and VS conditions, which were largest in iA1 and centred on more rostral locations. The peak voxels of the CM NBR were all located in slices 10–14 mm (Table 4) which showed strong similarity to the peak location of the PBRs to A and VA at 14 mm (Table 3).

In the visual cortex, we observed bilateral CM NBR and strongly lateralized IM NBR. The greatest magnitude NBR was induced during the S and AS conditions, with much greater spatial extent and peak voxel *Z* > 12, whereas all other conditions showed peak voxel *Z* < 10 (Table 4). The CM NBRs showed the strongest magnitude in bilateral anterior regions but extended to central posterior areas and into lateral visual areas in the S and AS conditions. The CM NBR induced by the A condition showed a similar spatial pattern but was weaker in magnitude and restricted to central midline regions. The IM NBR showed two distinct posterior and anterior clusters (see the *z* = 0 mm column of Figure 2); the posterior cluster in iV1 mirrored the peak location of the PBR in cV1. However, the CM NBR formed one contiguous cluster that was strongest in bilateral anterior visual cortex. The difference in the spatial location of the IM and CM NBRs in the visual cortex was further shown by the peak voxel locations (Table 4), whereby the IM NBR peak response lay at *z* = −6 or −8 mm, showing close agreement with the PBR peak in the opposite hemisphere (Table 3). In comparison, the CM NBR showed similar magnitude responses to the IM NBR at the *z* = −6 and −8 level, but the CM NBR peak response was more dorsal at *z* = 14 or 16 mm.

Whilst the core regions of the DMN showed consistent NBR across all conditions, the spatial extent and magnitude of the response varied considerably. The largest factors in the DMN NBR variation were the presence of adjacent IPS activation during the V conditions and the strength of the response to S stimuli. The DMN showed the strongest magnitude NBR during the S and AS conditions (*Z* = 16.7–18.5) and the weakest NBR in the V and VA conditions (*Z* = 8.8–11.4) (Figure 2 and Table 4). In the S and AS conditions, the NBR extended beyond the PCC and IPL into the surrounding parietal cortex. The extent of frontal mPFC NBR was broadly similar across conditions.

#### 3.2.4 Spatial Overlap of NBR With PBR

**FIGURE 3:**
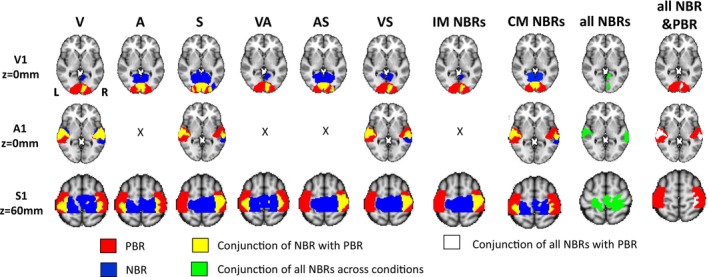
Summarising the spatial overlap of NBRs with each other and with the PBR. Upper, middle and lower rows show axial slices though primary visual, auditory and sensorimotor cortices. Shown in red is the group level PBR mask to V, A, and S stimuli respectively. Regions of conjunction between PBR and NBR are shown in yellow for each condition separately and also grouped across all IM and CM instances. NBR regions with zero overlap with PBR are shown in blue. To better illustrate overlap of NBR with potentially activated regions, the contralateral visual and sensorimotor PBR was reflected in the x = 0 axis in order to represent the bilateral PBR such as would occur during bilateral stimulation. Columns 1–6 show the group‐level conjunction of the PBR with the NBR to each stimulus condition. Column 7 shows the conjunction of all IM NBRs and their overlap with the PBR. Column 8 shows the conjunction of all CM NBRs and their overlap with the PBR. Column 9 shows the conjunction of all NBRs (green). Column 10 shows the conjunction of all NBRs with the PBR (white). X denote instances during auditory stimuli where no IM NBR was seen due to bilateral PBR in A1.

The spatial conjunction of the sensory NBR and PBR was computed to quantify the consistency of NBR regions across stimulus conditions and the extent to which NBR occurred in the same regions that showed PBR. Figure 3 displays the group level spatial overlap of each NBR with the PBR in each of the visual, auditory, and sensorimotor cortices, as well as the conjunction of all NBRs. We further summarize these results using Dice coefficients (DC) and % overlap metrics in Table 5.

**TABLE 5:** Metrics summarising the spatial overlap between PBR and NBR in primary visual (V1, rows 1 and 2), auditory (A1, row 3) and sensorimotor (S1, rows 4 and 5) cortices.

Overlap ROIs	V NBR	A NBR	S NBR	VA NBR	AS NBR	VS NBR	IM NBR	CM NBR
V1 PBR	—	0.43/49%	0.62/51%	—	0.51/47%	—	—	0.42/50%
V1 PBR reflected	0.36/83%	0.31/33%	0.54/46%	0.3/81%	0.52/46%	0.38/82%	0.28/89%	0.34/35%
A1 PBR	0.74/54%	—	0.47/53%	—	—	0.56/53%	—	0.53/52%
S1 PBR	0.28/16%	0.1/7%	—	0.17/11%	—	—	—	0.16/8%
S1 PBR reflected	0.18/11%	0.13/9%	0.37/24%	0.10/8%	0.4/25%	0.35/22%	0.36/24%	0.12/7%

*Note:* The first number shown is the dice coefficient, the second is the % of NBR voxels that overlapped with the PBR. Columns 1–6 show metrics for the PBR overlap with NBR to each of the conditions. Columns 7 and 8 show metrics for the conjunction of PBR with all of the IM NBRs; and PBR with all of the CM NBRs respectively. Grey shaded cells show CM NBR, white shaded cells show IM NBR. (—) Indicate no possible overlap with PBR.

In the visual cortex (Figure 3 upper row) the IM NBRs all showed very high spatial conjunction with the reflected PBR in right V1 (DC 0.3–0.38, % overlap > 80%, 2nd row Table 5), whilst the CM NBRs showed bilateral overlap with the PBR that was strongest in the S condition (DC = 0.62). The percentage overlap was lower for the CM NBR because the CM NBR had a much larger spatial extent into anterior V1 than the IM NBR.

In the auditory cortex (Figure 3 middle row) we observed a substantial overlap between the PBR to the A condition and the CM NBRs bilaterally, with the largest overlap observed with the V NBR (DC = 0.74, Table 5). The auditory cortex showed the largest overlap of any CM NBR with PBR (all > 50%).

In sensorimotor cortex (Figure 3 lower row) during all conditions, NBR was observed in a midline region that did not show PBR to the S conditions, leading to lower overlap metrics than for other cortices (Table 5). Only the most lateral of the NBR regions overlapped with the PBR; however, this was the hand area of the primary somatosensory cortex, which contained the peak response voxels, and as can be seen from Figure 3 NBR extended over a large proportion of the reflected PBR in right S1. CM NBR overlapped with PBR in bilateral S1 regions, with the V condition showing the largest (DC = 0.18, Table 5). Amongst the IM NBR, the spatial overlap with the reflected PBR was similar, DC = 0.35–0.4, 24%–25%.

The degree of spatial overlap with the PBR was larger for IM NBR than CM NBR in both visual (89% vs. 35%) and sensorimotor (24% vs. 7%) cortex, further suggesting that the IM and CM NBR occur in overlapping yet slightly different regions. This observation is further supported by the data in Table 6. Here we specifically compared IM with CM NBR locations. Auditory cortex NBRs showed a very high degree of spatial overlap across all instances (> 90%). The IM NBR induced in sensorimotor cortex all showed a very high spatial conjunction with each other (> 90%). While the CM NBRs in somatosensory cortex showed more variable conjunction overall, 77% of the IM NBR overlapped with the CM NBR. A similar pattern was observed in visual cortex, with a consistently high spatial conjunction (> 60%) between IM NBRs, but more variable conjunction between CM NBRs. Overall, 45% of the IM NBR overlapped with the CM NBR, further evidencing how the CM NBRs tended to occur primarily in anterior midline regions of visual cortex, whereas the IM NBR showed stronger responses in posterior ipsilateral V1.

**TABLE 6:** Metrics summarising the spatial overlap between IM NBR and CM NBR in primary visual (V1), auditory (A1) and sensorimotor (S1) cortices.

Overlap ROIs	IM vs. IM			CM vs. CM			IM vs. CM
V1	V vs. VA	V vs. VS	VA vs. VS	A vs. S	S vs. AS	A vs. AS	V/VA/VS vs. A/S/AS
	0.57/64%	0.80/67%	0.75/65%	0.72/49%	0.9/94%	0.8/56%	0.55/45%
A1	—	—	—	V vs. S	V vs. VS	S vs. VS	—
	—	—	—	0.53/94%	0.64/95%	0.86/96%	—
S1	S vs. AS	S vs. VS	AS vs. VS	A vs. V	V vs. VA	A vs. VA	S/AS/VS vs. V/A/VA
	0.94/95%	0.95/94%	0.94/94%	0.67/60%	0.78/92%	0.74/74%	0.59/77%

*Note:* The first number shown is the dice coefficient, the second is the % of the first listed ROIs voxels that overlap with the second listed ROIs voxels, for example, for cell 1 the % of NBR to V voxels that overlapped with the NBR to VA voxels. Columns 1–3 show metrics comparing different instances of IM NBR. Columns 4–6 show metrics comparing different instances of CM NBR. Column 7 shows metrics for the conjunction of all of the IM NBR and all of the CM NBRs respectively. Grey shaded cells show CM NBR, white shaded cells show IM NBR comparisons. (—) Indicate no possible overlap.

#### 3.2.5 Comparing PBR and NBR Between Single‐ Versus Dual‐Modality Conditions

**FIGURE 4:**
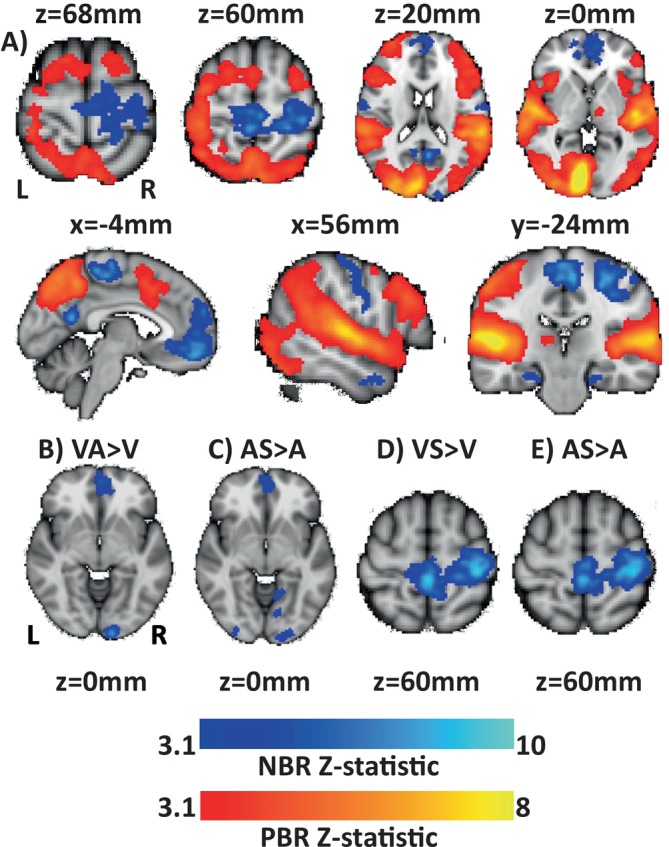
Group contrasts exploring regions where the magnitude of PBR or NBR was greater during dual than single modality stimulation conditions. Shown are (A) the contrast of all dual versus all single [VA + AS + VS] > [V + A + S]; and specific instances in visual cortex (B) VA>V; (C) AS>A and somatosensory cortex D) VS>V; (E) AS>A where the magnitude of NBR was greater during dual than single modality stimulation conditions. Significant regions are shown for PBR (red‐yellow) and NBR (blue). PBR *Z*‐statistics were calculated with mixed effects, NBR *Z*‐statistics were calculated with fixed effects, all cluster corrected at *p* < 0.05, *Z* > 3.1.

We contrasted dual versus single conditions to investigate whether NBR magnitude was modulated by the addition of a second stimulus. In general, dual modality conditions induced PBR and NBR in highly comparable regions to single modality conditions, but some notable differences in response amplitude were observed. Figure 4A shows the regions where PBR and NBR magnitude were significantly stronger during all dual conditions than during all single conditions (calculated by the contrast [VA + AS+VS] > [V + A + S]). PBR was significantly stronger in dual than single conditions primarily in cortical regions, including all three primary sensory cortices, along with the parietal, insula, prefrontal, and anterior cingulate cortex. There were no brain regions where PBR was stronger in single than in dual stimulation. Stronger magnitude NBR during dual than single conditions was observed in the midline and ipsilateral sensorimotor cortex, as well as small regions of ipsilateral V1, along with the mPFC and PCC DMN regions (Figure 4A). Figure 4B–E shows further examples derived from specific single vs. dual comparison GLMs. Regions of greater magnitude iV1 NBR were observed during VA than V (Figure 4B) and during AS than A (Figure 4C), showing that the magnitude of both IM and CM NBR in visual cortex was increased by dual stimulation. Furthermore, the sensorimotor cortex NBR showed greater magnitude during VS than V (Figure 4D) and during AS than A (Figure 4E), showing again that in sensorimotor cortex the IM NBR was stronger than the CM NBR. These were the only instances observed of significantly greater response during dual than single stimulation; for example, no difference in iV1 NBR was observed during VS compared to V and no difference in iS1 NBR was observed during VA compared to V or A, and no difference in A1 NBR was observed during VS compared to S.

#### 3.2.6 PBR‐NBR and NBR‐NBR Relationships

**FIGURE 5:**
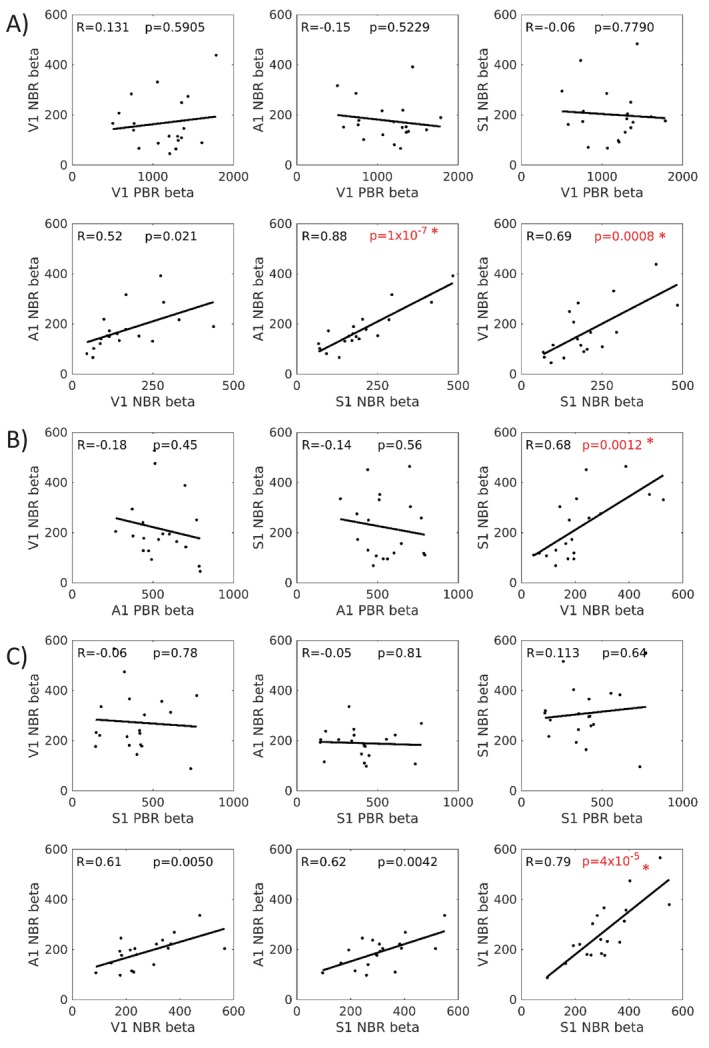
Linear correlation plots of PBR‐NBR and NBR‐NBR relationships shown for Visual (A), Auditory (B) and Somatosensory (C) stimulation conditions. Therefore in (A) V1 NBR and V1 PBR are the IM responses and all others are CM responses. No significant relationships were observed for any PBR and NBR combination. However, several significant (*p* < 0.003, adjusted for 15 multiple comparisons) positive NBR‐NBR relationships were observed, indicated by red font and *, as well as a number of trends.

**FIGURE 6:**
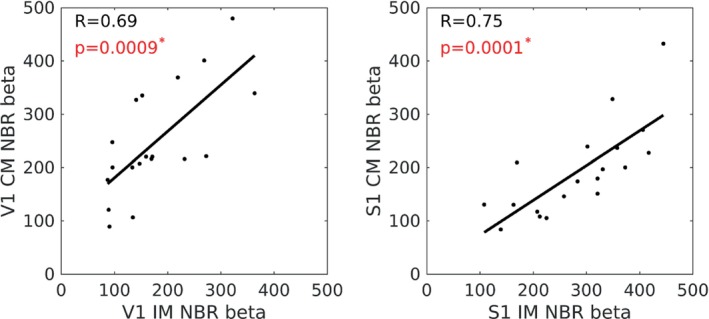
Linear correlation plots of IM‐NBR versus CM‐NBR relationships shown for visual (A) and sensorimotor (B) cortex. NBR responses were measured as the peak beta‐weight in each individual. IM‐NBR responses were calculated as the mean of all IM‐NBRs: For V1 from the conditions V, VA, and VS; for S1 from the conditions S, AS, and VS. CM‐NBR responses were calculated the mean of all CM‐NBR: For V1 from the conditions A, S, and AS; for S1 from the conditions V, A, and VA. Significant (*p* < 0.05, positive NBR‐NBR relationships were observed, indicated by red font and *.

Finally, we computed linear correlations between subject's peak beta weights of PBR and NBR. Here we studied relationships between the magnitude of BOLD responses induced in different brain regions concurrently by the same stimulus, or between the magnitude of BOLD responses induced in the same brain region but at different times by different stimuli (Figure 5). We found no significant correlations between PBR and any NBR. However, we did observe multiple relationships between NBRs. Figure 5 shows the lack of relationship between PBR and NBR for visual (5A), auditory (5B) and somatosensory (5C) stimuli (all *p* > 0.45). In contrast, all seven of the NBR‐NBR relationships showed positive correlations, with four showing highly significant correlations that passed a *p* < 0.003 threshold Bonferroni corrected for multiple (15) comparisons, and two others showing very strong trends. The four significant correlations were: S1‐A1 NBR and S1‐V1 NBR during V stimuli; V1–S1 NBR during A stimuli and S1‐V1 NBR during S stimuli. Qualitatively very similar results were also observed in the equivalent plots for dual stimuli, but for brevity we omit them here as they do not provide any additional insight. Linear correlations between the peak beta weight of mean IM‐NBR and mean CM‐NBR were separately studied for visual and sensorimotor cortices (Figure 6). Figure 6 shows strong positive correlations between IM and CM NBR in both cortices (*R* > =0.69, *p* < 0.001), showing that subjects with high magnitude IM NBR also displayed high magnitude CM NBR.

## 4 Discussion

This study provides the first comprehensive investigation and comparison of both intra‐modal (IM) and cross‐modal (CM) NBR to lateralised (right) stimulation of the three primary sensory modalities: visual, auditory, and somatosensory. Participants were cued to perform a target detection task during 6 s periods of either single‐ or dual‐sensory stimulation. All conditions induced NBR in the DMN as well as CM NBRs in the sensory cortex of all unstimulated, “non‐task‐relevant” modalities. IM NBRs were observed in the ipsilateral visual and sensorimotor cortex during all visual and sensorimotor stimuli.

Our primary findings were that: NBRs occurred in all unstimulated sensory cortical areas regardless of modality; NBR in a given cortex occurred with broadly comparable spatial extent and magnitude between conditions but also showed some small stimulus‐specific differences; IM and CM NBR induced by different stimuli showed close spatial agreement; all NBRs showed considerable spatial overlap with regions of PBR, but the conjunction was higher for IM than CM NBR; IM NBR induced by somatosensory stimulation showed the strongest magnitude and spatial extent; PBRs in sensory and fronto‐parietal areas were significantly stronger in dual than single conditions, whilst for NBR only small regions of visual and sensorimotor cortex showed increased magnitude response during dual conditions; significant linear correlations were observed between the magnitude of IM and CM NBR. We now discuss our findings in more detail with respect to the five research questions that motivated this work.

### 4.1 Do NBR Occur Similarly in Unstimulated Sensory Cortex During Different Sensory Stimuli?

Previous work on CM‐NBRs has focused on those induced in the auditory cortex by visual stimuli and vice versa (Hairston et al. 2008; Laurienti et al. 2002; Mozolic et al. 2008). In addition to these responses, we report that CM NBRs are also induced in the bilateral sensorimotor cortex and that somatosensory stimuli induce both visual and auditory CM NBRs. To our knowledge, this is the first demonstration that CM NBRs are a consistent and mutual response between all modalities and indicative of a fundamental mechanism to suppress activity in all non‐task‐relevant sensory cortices, potentially reflecting an fMRI measure of the neural hyperpolarizations reported previously in rodents (Iurilli et al. 2012).

Consistent with previous work (Yetkin et al. 2004) we observed bilateral auditory cortex PBR (Figure 2) during unilateral auditory stimuli, and therefore no IM auditory NBR. This is due to each primary auditory cortex processing stimuli from both contralateral and ipsilateral ears because, unlike the visual and somatosensory pathways where all fibers cross and ascend the contralateral pathway, approximately 25% of cochlear neurons synapse on the ipsilateral superior olive and ascend the ipsilateral pathway (Mather 2016), resulting in bilateral A1 activation.

### 4.2 How Do the Spatial Locations of IM and CM NBR Compare to That of the PBR and to Each Other?

Whilst there was a high degree of consistency in the spatial location, extent, and magnitude of IM NBR and CM NBR across conditions (Figure 3 and Tables 5 and 6), and generally a high degree of overlap with PBR regions, some notable differences included that: (1) IM NBRs were more spatially specific in that they were strongly lateralised, whilst CM NBRs were bilateral. CM NBRs spatially overlapped with the IM NBR regions and extended into additional areas, for example, contralateral sensory cortex and more extensively within ipsilateral visual cortex; (2) regardless of cortex, NBRs were highly consistent within IM or CM occurrences, that is, the magnitude and extent of IM NBR in visual/somatosensory cortex was highly comparable across all conditions containing visual/sensorimotor stimuli; (3) Whilst S1 NBRs occurred in very similar locations regardless of the stimulus, A1 NBRs were the strongest and most bilateral during V stimuli, being more lateralised to the right and more rostral in locations in conditions including S stimuli; in visual cortex, IM NBRs to all conditions that included V stimuli showed peak responses in posterior iV1 closely opposite the PBR peak. Whereas the CM NBR to A, S, and AS stimuli was more anterior and superior.

The visual and sensorimotor cortices displayed both IM and CM NBR. Across conditions these arose in broadly comparable areas with very similar peak voxel locations but with notable differences in their magnitude or spatial extent (Figure 2 and Table 4). The extent of the IM NBR was reduced by the presence of the contralateral PBR but even allowing for that, the spatial pattern of the NBR in ipsilateral cortex was different between IM and CM instances (Figure 2). In visual cortex, the CM NBR formed a contiguous midline cluster, extending from anterior into posterior V1 and closely resembling the central visual network commonly reported in resting state data (Luca et al. 2006). Whereas the IM NBR comprised distinct anterior and posterior iV1 clusters. In sensorimotor cortex, the IM NBR was strongly focused around its peak in iS1 with extension into midline S1 regions, whereas the CM NBR showed a bilateral pattern with distinct left and right hemisphere clusters. Overall we observe that CM NBRs were highly similar in spatial location despite being induced by very different stimuli which suggests that CM NBR occurs not via a sensory specific pathway but by a more general mechanism. Furthermore, the small spatial inconsistencies between IM and CM NBR may indicate that they can arise from, at least partially, different generative mechanisms.

### 4.3 How Do the Magnitudes of the IM and CM NBR Compare?

Whilst NBRs were broadly consistent, we observed condition‐level nuances, and it was not the case that stimulation of a given modality induced identical NBRs in all other sensory regions. Primary sensory cortex PBRs were very similar in magnitude across IM and CM conditions, so differences in NBRs seem unlikely to be explained solely by differences in PBR.

The strongest NBRs were those observed in the sensorimotor cortex during conditions that included S stimuli, whereas across the other conditions, the NBR magnitudes were broadly comparable. It is unclear whether this difference in NBR magnitude is specific to sensorimotor cortex responses or whether it could occur due to differences in stimulation type, as MNS is a particularly arresting, slightly uncomfortable physical stimulus compared to visual images and auditory tones to which participants are more familiar. Equating the bottom‐up input and saliency between stimuli of different sensory modalities is difficult, and whilst the possibility that MNS is a more “deactivating stimulus” is supported by it also inducing the greatest DMN NBR, we note that the cS1 PBR induced in S conditions was no larger in magnitude than the other primary sensory PBRs.

### 4.4 What Is the Relationship Between NBRs, Do Subjects That Show Strong IM NBR Also Show Strong CM NBR?

We found no relationship between the magnitude of the PBR and the magnitude of any NBR but did observe significant positive linear correlations for IM vs. IM NBRs, IM versus CM NBRs, and CM versus CM NBRs. This showed that NBR relationships occurred to the same stimulus in different regions as well as between NBRs to different stimuli in the same region (Figures 5 and 6). Given the number of PBR and NBR combinations, there was a considerable multiple comparisons problem, but the correlations were significant after stringent Bonferroni correction. We feel that observing no PBR‐NBR correlations whilst observing multiple NBR‐NBR correlations presents a telling contrast and indicates that although there is no apparent link between activation and deactivation, the participants that showed the strongest NBR to one condition also showed the strongest NBRs to other conditions, and vice versa. This finding may represent a physiological or behaviourally consistent mechanism across conditions whereby some participants were able to perform the task with minimal suppression of non‐task‐relevant regions (small NBR), whilst others showed more widespread suppression and NBRs. To explore this, we tested for relationships between subjects' behavioural accuracy scores and the magnitude of IM NBR but found no significant correlation in any condition (all *p* > 0.15).

### 4.5 Do Dual Modality Stimuli Cause Greater Magnitude NBR Than Single Modality Stimuli?

Whilst we cannot conclude that dual stimulation had an overall effect in enhancing NBR in all instances, as there was no effect in the auditory cortex, we found it was possible for NBR to show increased magnitude during dual conditions, especially in the sensorimotor cortex.

We observed some modality effects. In the sensorimotor cortex, IM NBRs appeared to dominate over CM NBRs, as was shown by the larger NBR magnitude during VS and AS than during either V or A conditions. However, the addition of a CM stimulus (A or S) did cause an increase in the NBR in the visual cortex compared to that induced by A or V alone. Furthermore, as expected, we found that PBRs dominated NBRs in that sensory stimulation caused PBRs irrespective of whether that modality was task‐relevant. For example, the VA condition showed only PBRs in the auditory cortex during attend‐V trials. Therefore, PBRs were determined primarily by the bottom‐up stimulus input.

It is notable that the increases in NBR during dual stimulation were relatively small and spatially focused, whereas the increases in PBR during dual stimulation were substantial and widespread (Figure 4). Therefore, despite a large increase in the brain's activation, only a small increase in the magnitude of deactivation was seen. Dual conditions resulting in greater neural recruitment and metabolic expenditure across the whole brain are unsurprising due to the increased stimulation and attentional demands required to perform the task. However, a proportionate increase in NBR magnitude was not fully observed despite the additional activation. This suggests that the additional demands of the dual task could be met largely through extra activation, with a smaller contribution from additional inhibition.

The observation of increased magnitude NBR during each of the dual conditions suggests these differences are not modality specific and are unlikely to arise purely from differences in the stimulus input. This enhanced magnitude NBR could arise due to a combination of inhibitory effects due to multiple sensory inputs, or further withdrawal of attention away from the remaining unstimulated brain regions in order to facilitate processing in more task‐relevant areas (Hairston et al. 2008). Our data are partly consistent with this, although the increase in NBR during dual conditions is relatively small despite moving from single (e.g., V) to dual (e.g., VA attend V) conditions being associated with a substantially increased task demand due to the increased need to suppress distraction and interference from the non‐target modality (e.g., A) and increased withdrawal of attention away from the non‐task‐relevant modality (e.g., S). If NBR were predominantly driven by such changes in the allocation of attention, then a larger modulation of NBR that was also more consistent across all dual conditions would have been expected. Although a counter‐argument to this would be that there may be a floor effect present, as a maximum level of deactivation would be reached once the prestimulus level of activity is entirely suppressed. In some circumstances, the sensory stimuli could drive the NBR close to maximum magnitude, resulting in minimal further modulation by attention being possible.

These findings add to previous work that investigated NBR summation in the visual cortex due to combinations of different visual stimuli (Wilson et al. 2020). In that study, the authors report no enhancing effect upon the NBR by multiple stimuli, which they interpret as evidence that individual stimuli each exerted a separate inhibitory effect on non‐stimulated regions; but once delivered in combination, these effects operated as a binary system. However, they used a passive task with minimal cognitive demand and therefore little requirement for suppression of non‐task‐relevant regions. Previous work has shown that the amplitude of both visual and somatosensory IM NBR increased with stimulus intensity and/or duration (Kastrup et al. 2008; Shmuel et al. 2002). Furthermore, DMN NBR magnitude has also been observed to increase with task difficulty (McKiernan et al. 2003; Singh and Fawcett 2008). This evidences that NBR magnitude can scale even in passive tasks, but also that the experimental context is important and that changes in either the bottom‐up stimulus input or the level of top‐down control are required, such as in the present study, where performing a difficult target detection task would benefit from the suppression of any distracting or interfering input from other senses.

### 4.6 Potential Origins and Functional Relevance of NBR

Uncertainty has long surrounded the origins of NBR, but the extent to which NBR arises from neurometabolic versus vascular sources is relatively well studied compared to the question of the generative network mechanisms that contribute to and modulate NBR magnitude.

Theories of blood steal originated in visual cortex studies and rely heavily on NBR having close spatial proximity to, and sharing the same vascular supply as the activated regions. Doubt has already been cast on this as ipsilateral NBRs arise in the opposite hemisphere to the PBR, which comprises a different vascular territory (Kastrup et al. 2008; Mullinger et al. 2014; Schafer et al. 2012; Kastrup et al. 2008; Mullinger et al. 2014; Schafer et al. 2012). The possibility of such vascular mechanisms underlying cross‐modal NBR seems unlikely unless long‐range neural signals can regulate blood supply concurrently in multiple sensory networks. However, intracellular recordings in mice have shown that activation of the auditory cortex by a noise burst resulted in localized GABA‐ergic inhibition in supragranular pyramidal cells of the visual and somatosensory cortices (Iurilli et al. 2012). Such mechanisms could underlie the cross‐modal NBRs observed in the present study.

NBR may arise primarily for reasons of metabolic efficiency, whereby energy is conserved by reducing net excitation in non‐task‐relevant regions (Lauritzen et al. 2012), as evidenced by decreases in oxygen metabolism in NBR regions (Mayhew et al. 2014; Schafer et al. 2012; Shmuel et al. 2002) and decreases in the concentration of glutamate that correlate with the NBR amplitude (Martinez‐Maestro et al. 2019). This view is supported by the common observation of NBR during entirely passive tasks (Klingner et al. 2010; Mullinger et al. 2014; Shmuel et al. 2002; Shmuel et al. 2006; Wade and Rowland 2010; Wilson et al. 2019; Wilson et al. 2020) where there is minimal benefit to task performance arising from the suppression of distracting sensory input. However, some evidence suggests that NBR may also directly benefit task performance (Amedi et al. 2005; Bressler et al. 2007; Hairston et al. 2008) possibly arising via attentional mechanisms (Corbetta and Shulman 2002; Gilbert and Li 2013; Paneri and Gregoriou 2017) that help to prioritize processing of a spatial location or sensory modality, while information originating from non‐task‐relevant regions is suppressed to minimize potential distraction or interference. Mozolic et al. (2008) suggested that the benefit of attentional allocation to performance arises not only from an increase in PBR magnitude but also from modulation of the NBR (Mozolic et al. 2008). Therefore, NBR could present a neurophysiological marker of such processes, a functional inhibition that can be modulated by task input and demands.

We observed robust IM and CM NBR in all stimulus conditions that were slightly increased during dual stimulation. Given that increases in NBR during dual stimuli were relatively small compared to the overall size of the NBRs and the effects of attention on BOLD responses are generally smaller than those of stimulus inputs, we suggest that the enhanced NBR during dual trials is consistent with a strong contribution from bottom‐up input that was further modulated by attention during dual conditions. Although no significant differences in accuracy were observed between single and dual conditions, this does not prove that single and dual conditions were equivalent in terms of behaviour and brain processing. For instance, differences in reaction time are often observed between conditions without differences in accuracy (Mazaheri et al. 2014; van Ede et al. 2012). It is possible that this would be the case in our task, but we are unable to study this as our delayed response period did not enable us to measure reaction times.

These findings suggest that NBR is driven by a combination of bottom‐up and top‐down influences whereby both feed‐forward signals from subcortical or activated sensory regions (Logothetis et al. 2010) and feed‐back mechanisms such as higher‐level attentional control (Lauritzen et al. 2012) are exerted to suppress activity in low‐level sensory regions that are not required by the task. It is likely that the dominant factor in NBR, or other BOLD signal modulation, depends on the behavioural situation. Whilst passive sensory stimulation induces strong NBR in conditions with minimal attentional requirement, the observation of auditory and somatosensory cortex NBR during visual imagery (Amedi et al. 2005) suggests that feedforward input is not necessary for NBR to occur. This is further evidenced by studies showing that shifts in attention, either within or between modalities, can induce NBR in the absence of sensory stimulation (Mozolic et al. 2008; Tootell et al. 1998). Thus, some instances of NBR may reflect the task‐induced removal of afferent inputs that are present during the resting state. Finally, it must be remembered that experimental manipulations may not influence PBR and NBR in a comparable manner as evidence accumulates that the two responses may arise from different neuro‐vascular coupling mechanisms (Devi et al. 2022; Huber et al. 2014; Mullinger et al. 2014).

### 4.7 Limitations and Future Work

NBR in the ipsilateral visual regions was the weakest NBR observed. Inspection of individual subject 2nd level results showed some instances of visual PBR occurring bilaterally in three subjects, suggesting that they moved their gaze to follow the unilaterally presented checkerboard rather than centrally fixating as instructed. We therefore attribute the weak ipsi V1 NBR to the poor behaviour of a few participants rather than a fundamental difference in the physiology of this response. Future work should include eye‐tracking measurements to quantify such effects more clearly and allow rejection of trials featuring incorrect eye movements. Future work should consider alternative paradigms that create the need for suppression of a multisensory stimulus. Using more ecologically valid stimuli may create greater conflict between sensory inputs and an increased need for distractor suppression, such as performing visual motion perception while ignoring motion‐related sounds or physically moving tactile stimuli. Or integrated multisensory stimulation, where trials could be contrasted between both or only one modality containing task‐relevant information, with the other providing interference.

## 5 Conclusion

This study has increased current understanding of an important component of brain function, the negative BOLD response, by studying the consistency of IM, CM, and DMN NBR between combinations of visual, auditory, and somatosensory stimulation and how their magnitude was modulated by the modality and the number of sensory stimuli. Our findings reflect an apparently close functional correspondence whereby specific sensory cortical areas were activated according primarily to the bottom‐up drive of the afferent stimulus, whilst activity in all other sensory regions was suppressed. When stimulation occurred in a different sensory modality, the region previously activated was suppressed instead. Correlations between different instances of NBR across subjects indicated interesting inter‐individual variability in the expression of the response. We found that additional sensory stimuli created an attentional modulation whereby NBR magnitude was further enhanced, although it remains unclear whether this provides an actual functional benefit to task performance or simply reflects a metabolic energy‐saving mechanism.

## Data Availability

The data that support the findings of this study are available from the corresponding author upon reasonable request.
